# Factors associated with deaths by tuberculosis in the state of Mato Grosso, 2011-2020: retrospective cohort study

**DOI:** 10.1590/S2237-96222024V33E20231402.EN

**Published:** 2024-11-11

**Authors:** Vanessa da Silva Lopes, Rodrigo de Macedo Couto, Alexandre Peron da Luz, Pãmela Rodrigues de Souza Silva, Jaqueline Costa Lima

**Affiliations:** 1 Universidade Federal de Mato Grosso, Faculdade de Enfermagem, Cuiabá, MT, Brasil; 2 Universidade Federal de São Paulo, Departamento de Medicina Preventiva, São Paulo, SP, Brasil; 3 Secretaria de Estado de Saúde, Cuiabá, MT, Brasil

**Keywords:** Tuberculosis, Deaths, Risk Factors, Retrospective Cohort Study, Tuberculosis, Mortalidad, Factores de Riesgo, Estudio de Cohorte Retrospectivo

## Abstract

**Objective:**

To investigate factors associated with tuberculosis deaths in Mato Grosso state, Brazil, from 2011 to 2020.

**Methods:**

Retrospective cohort study with data obtained from the Notifiable Health Conditions Information System and the Mortality Information System. Deaths were qualified using probabilistic linkage and analyzed using Poisson regression.

**Results:**

12,331 cases and 525 deaths were identified over 10 years. The factors associated with death were: age ≥60 years (RR: 7.70; 95%CI 1.91;31.04), incomplete elementary and high school education (RR: 3.66; 95%CI 1.34;9.96), illiteracy (RR: 4.50; 95%CI 1.60;12.66), homeless population (RR: 2.41; 95%CI 1.34;4.35), alcohol use (RR: 1.45; 95%CI 1.04;2.02), male sex (RR: 1.48; 95%CI 1.04;2.09) and tobacco use (RR: 1.32; 95%CI 0.98;1.77). Laboratory confirmation was a protective factor.

**Conclusion:**

Risk of death was higher in men over 60 years old, with low education levels, in vulnerable situations, and who used alcohol/tobacco.

## Study contributions

**Main results:**

Risk of death was higher in the elderly, males, people with low education levels, homeless people, alcohol and tobacco users. Laboratory confirmation was a protective factor.

**Implications for services:**

Raising awareness of health professionals regarding risk factors, especially regarding risk behaviors and laboratory confirmation of tuberculosis, to which efforts should be targeted.

**Perspectives:**

It would be strategic to study survival in order to assimilate the effect of time and to study people with drug-resistant tuberculosis in order to update treatment recommendations. Health service managers need to define public policies aimed at the determinants found.

## Resumen

**Objetivo:**

Investigar los factores asociados a la mortalidad por tuberculosis en Mato Grosso, entre 2011/2020.

**Métodos:**

Cohorte retrospectivo con datos obtenidos del Sistema de Información de Enfermedades de Declaración Obligatoria y Sistema de Información de Mortalidad. Las muertes se calificaron mediante relación probabilística y se analizaron por regresión de Poisson.

**Resultados:**

En 10 años se identificaron 12.331 casos y 525 muertes. Los factores asociados a muertes fueron: edad ≥ 60 años (RR: 7,70; IC_95%_ 1,91;31,04), educación primaria/secundaria incompleta (RR: 3,66; IC_95%_ 1,34;9,96), analfabetismo (RR: 4. 50 IC_95%_ 1.60;12.66), población sin hogar (RR: 2.41; IC_95%_ 1.34;4.35), alcohol (RR: 1.45; IC_95%_ 1.04;2.02), sexo masculino (RR: 1.48; IC_95%_ 1.04 ;2,09), tabaco (RR: 1,32; IC_95%_ 0,98;1,77). La confirmación de laboratorio fue un factor protector (RR: 0,68; IC_95%_ 0,52;0,93).

**Conclusión:**

Hubo mayor riesgo de muerte en hombres mayores de 60 años, con baja escolaridad, en situación de vulnerabilidad, que consumen alcohol/tabaco.

## INTRODUCTION

Tuberculosis is an infectious and communicable disease, the main etiological agent of which is *Mycobacterium tuberculosis*, which affects the lungs and other organs. It occurs mostly in developing countries, having a close relationship with populations in disadvantaged social, economic and cultural conditions, that is, living in poverty.^1^ ^)-(^ ^3^

Tuberculosis is considered to be a global health problem and, despite being preventable and curable, it is one of the main causes of death worldwide, surpassing AIDS and malaria. ^(^ ^1^ In Brazil alone, which is among the 30 countries with the highest number of cases, approximately 78,000 new cases were reported in 2022, and 5,000 tuberculosis deaths in 2021. ^(^ ^4^ Reducing these numbers is in particular the target of interest of the National Plan to End Tuberculosis as a Public Health Problem, which establishes a 90% reduction in the incidence rate and a 95% reduction in the number of tuberculosis deaths, both compared to the rates reported for 2015. ^(^ ^1^ ^),(^ ^4^

The scenario described reflects the persistence of tuberculosis as an important and challenging problem in the context of the population’s health and, considering the social and multifactorial production of the disease, ^(^ ^5^ its mortality determinants are conditioned by different characteristics of demographic, cultural and development dimensions. Some studies have been concerned with analyzing and describing these factors in various states and regions of Brazil, providing proof of their variation in degree of relevance and occurrence according to the characteristics of the research location. ^(^ ^6^ ^),(^ ^7^

However, scientific production on this topic in Mato Grosso is incipient and limited, with ecological studies carried out in specific priority municipalities, such as Rondonópolis and Barra do Garças, or broader analyses of the Midwest region as a whole being more common. ^(^ ^8^ ^)-(^ ^10^ Despite having a Human Development Index (HDI) considered high (0.736), ^(^ ^11^ the state still has high tuberculosis endemicity and mortality, having recorded an incidence rate of 31.8 cases per 100,000 inhabitants in 2022 and a mortality rate of 2.0 deaths per 100,000 inhabitants in 2021. ^(^ ^4^ These figures reveal a scenario that seems incongruous and that may indicate the need to gain knowledge of the specific determinant factors associated with deaths that affect this population. 

Identification of these factors, enabled in particular by this investigation, is essential for intensifying preventive measures and health service management throughout Brazil, since, with more specific data, it is possible to provide input and support for government strategies for developing public health policies that are more adapted to local needs, in order to achieve the goals of the National Plan to End Tuberculosis as a Public Health Problem. 

The objective of this study was therefore to investigate the factors associated with tuberculosis deaths in Mato Grosso state, Brazil, from 2011 to 2020.

## METHODS

*Study design and location*

This is a retrospective cohort study of tuberculosis cases and the occurrence of death due to the disease in the territorial area of the state of Mato Grosso, Brazil, from 2011 to 2020, reported on the Notifiable Health Conditions Information System (*Sistema de Informação de Agravos de Notificação - SINAN*) and the Mortality Information System (*Sistema de Informação sobre Mortalidade - SIM*). The data were obtained in February 2023, using a removable data storage device, provided by the Epidemiological Surveillance Service of the Mato Grosso State Health Department. The information systems referred to above are national systems used by municipal health services throughout Brazil, which input data on health conditions and deaths which in turn form the state-level and national-level databases. Tuberculosis notification in Mato Grosso became more structured and efficient with effect from the late 1990s with the introduction of the SINAN, significantly improving the state’s monitoring and response capacity. ^(^ ^12^ ^)^

The state of Mato Grosso has 141 municipalities and is located in the Midwest region of Brazil, covering an area of 903,208.361 km². Its population, according to the most recent Census conducted in 2022, was 3,658,813 inhabitants. Its population density is 4.05 inhabitants/km^2^ and its HDI is high (0.736). ^(^ ^11^ According to the National Registry of Health Establishments, the state has services distributed across all levels of care, totaling 9,103 health establishments. Of these, 56% (n = 5,110) are outpatient service units, 12% (n = 1,113) primary health care centers - covering 85% of the population -, 8% (n = 745) diagnostic and therapy support service units, and some 2% (n = 166) are hospitals. There are 9,341 beds available for hospitalization, almost 73% (n = 6,778) of which are provided by Brazilian National Health System services. Of the total hospital beds, 31% (n = 2,896) are clinical (82% of these are medical/general clinical beds), 28% (n = 2,609) are surgical, 15% (n = 1,357) are complementary, that is, intensive care unit or intermediate care beds, and 27% (2,479) are for other specialties. ^(^ ^13^

*Participants*

The study population was made up of tuberculosis cases in people of both sexes aged 10 years or older. Those indicated as being in the “change of diagnosis” category found in variable number 62 of the tuberculosis monitoring form were excluded, as they were not tuberculosis cases. Those with case closure recorded as “transfer” (meaning patient referred to another service to continue treatment) and those who had case closure due to prescription of treatment regimens other than the basic regimen (according to Ministry of Health guidelines, these cases must be closed on the SINAN and reported on the Tuberculosis Special Treatment Information System instead). The number of cases in the state during the study period, submitted to these criteria, determined the sample size, the outcome of which was tuberculosis deaths and non-deaths. 

*Variables, data source and measurement*

The following variables were obtained from the SINAN: 

a) sociodemographic variables: sex (female and male); age categorized into age groups (10-17 years old, 18-39 years old, 40-59 years old and 60 years old and over); race/skin color (White and non-White, including Black, mixed race, Indigenous and Asian); schooling (complete higher education, complete elementary and high school education, incomplete elementary and high school education, illiterate); street dweller (yes and no); health professional (yes and no); immigrant (yes and no); and deprived of liberty (yes and no). 

b) clinical variables: treatment (new case and retreatment); observed treatment (yes and no); diabetes (yes and no); HIV (yes and no); and laboratory confirmation (no and yes). The latter variable was added to the analysis after grouping the variables relating to diagnostic tests (sputum smear microscopy, culture and rapid molecular test) into a single variable, so that any of the three tests having been carried out was considered sufficient to indicate that laboratory confirmation of tuberculosis had been performed.

c) behavior variables: alcohol use (yes and no); drug use (yes and no); tobacco use (yes and no).

Admission types, as per variable number 32 of the notification form (“new case”, “not known” e “after death”), were considered to be new cases, while “relapse” and “readmission after dropout” were recategorized as retreatment. “Deaths due to tuberculosis” were considered to be those recorded with tuberculosis as the underlying cause on the SIM, and “cure or non-death” were those corresponding to codes A15 to A19 of the International Classification of Diseases (ICD-10), in the period from 2011 to 2020. 

We decided not to use variables with a percentage greater than or equal to 50% of unknown or blank values in the final model. In the assessment carried out by the authors, no variables were identified as possible sources of collinearity.

*Statistical analyses*

Deaths recorded on the SIM were used to qualify the information recorded on the SINAN, regarding the variable “Case Closure Status”, field 62 of the monitoring form. Therefore, the probabilistic record linkage method was used, since the databases used did not contain the same identifier variable. To this end, we used RecLink III software (version 3.1.6.3160). This method is based on the joint use of common identification fields present in both databases, with the aim of identifying how likely it is that a pair of records correspond to the same individual. ^(^ ^14^ In the initial stage, using Stata software (version 16.1), the databases were prepared by standardizing the variables of interest (date of birth, patient’s name, mother’s name, sex and municipality of residence). 

Subsequently, we used RecLink III software to create logical blocks of records by means of a process consisting of three blocking stages. Algorithms were applied to perform an approximate comparison of character strings, taking into account possible phonetic and typing errors. This comparison was made based on information such as name, mother’s name and date of birth. Scores were calculated to summarize the general degree of agreement between records belonging to the same pair. In the last step, matching (combination) occurred to establish thresholds that classify pairs of records as true, mismatched or doubtful. The manual review of doubtful pairs was conducted by a single investigator, and, during this process, the reclassification of these pairs as true or not true followed tiebreaker criteria, such as the patient’s name, date of birth, mother’s name and municipality of residence. Pairs that remained doubtful after the manual review were classified as not belonging to the same individual, therefore adopting a more conservative approach.

Initially, we performed a descriptive analysis of the distributions of the characteristics of the population studied, presenting the data in tables with absolute numbers and percentages. Next, relative risks (RR) were estimated in order to estimate the magnitude of association between the exposure variable and death, indicating how many times the occurrence of death in those exposed is greater than that among those not exposed. The RRs were calculated by taking the ratio between the incidence of death in exposed people and the incidence of death in non-exposed people. ^(^ ^15^ Statistical significance was taken to be 5% and was tested using the chi-square test, as the variables had a categorical distribution. 

Finally, we used a Poisson regression model to analyze factors associated with the occurrence of death, taking death due to tuberculosis as the dependent variable and potential risk factors as independent variables. Estimates of crude and adjusted relative risks (RRs) were made with their respective 95% confidence intervals. Variables with a p-value < 0.20 in the crude analysis were selected for the final Poisson regression model. Sex and age group were included in the model as adjustment variables, based on the literature on the topic. Predictor variables were added to the model one at a time, starting with the predictor variable with the lowest p-value. This process continued until all selected predictor variables had been tested (forward method). Statistical significance (5%) of the variables in the models was assessed using the Hosmer-Lemeshow test. We used Stata version 16 to perform the statistical analysis. 

*Ethical aspects*

This study was approved on August 18th 2021 by the Research Ethics Committee of the *Universidade Federal de Mato Grosso* (Certificate of Submission for Ethical Appraisal No. 47657721.7.0000.8124) as per Opinion No. 4.915.563.

## RESULTS

From the initial total of 16,899 tuberculosis cases reported on the SINAN, for the 10-year period analyzed (2011-2020), the following were excluded: 86 duplicate records (0.5%); 633 changes of diagnosis (3.7%); 587 cases aged up to 9 years (3.5%); 2 records without gender identification (0.01%); 1,204 transfers (7.1%); 2,056 cases of drug resistance, treatment regimen changes and treatment failures (12.2%). As a result, 12,331 (73%) records remained. In the case of the SIM database, 8 deaths of cases aged up to 9 years were excluded (1.1%), leaving 717 deaths due to tuberculosis. 

**Figure 1:**
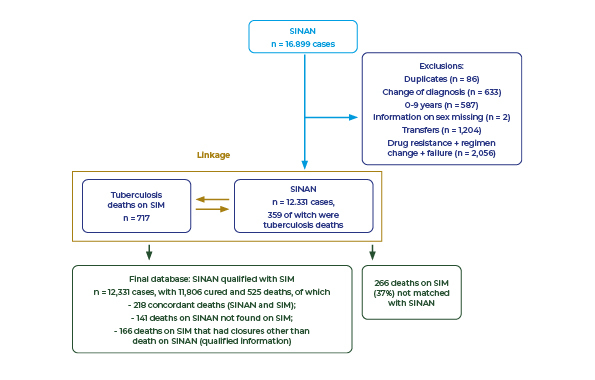
Flowchart of the probabilistic linkage between the Notifiable Health Conditions Information System (SINAN) and the Mortality Information System (SIM) databases, Mato Grosso, Brazil, 2011-2020

With the aim of qualifying deaths, a linkage technique was used between the SINAN and the SIM, by means of which 218 deaths were matched. Subsequently, 141 deaths held on the SINAN but not found on the SIM and 166 deaths held on the SIM but not found on the SINAN were added, totaling 525 deaths (Figure 1). At the end of the case closure qualification stage, 12,331 cases of tuberculosis were identified, of which 525 (4.26%) died.

The highest relative risk associated with death due to tuberculosis was related to the male sex (RR: 1.52; 95%CI 1.26;1.91) (Table 1). Regarding age group, risk was higher in the older age groups, presenting a “dose-response” effect, that is, the older the age group, the higher the risk of death (40 to 59 years old ‒ RR: 3.57; 95%CI 1.58;8.10; 60 years old or over ‒ RR: 7.86; 95%CI 3.47;17.80). Furthermore, high relative risk stands out among individuals who had incomplete elementary and high school education (RR: 3.69; 95%CI 1.95;6.99) or were illiterate (RR: 7.15; 95%CI 3.65;13.99). Regarding special populations, street dwellers had higher risk of death (RR: 2.45; 95%CI 1.49;4.49) (Table 1).

**Table 1:** Analysis of Relative Risks (RR) and respective Confidence Intervals (95%CI) of the demographic, socioeconomic and dwelling place characteristics of individuals and special populations with tuberculosis, by cases and deaths, Mato Grosso, Brazil, 2011-2020

Demographic, socioeconomic and housing characteristics	Total n = 12.331		Tuberculosis cases					
			Non-deaths n = 11.806		Deaths n = 525		RR (95%CI)^a^	p-value
	N	%	N	%	N	%		
Sex^b^								
Female	3.895	31,6	3.773	32,0	122	23,2	1,00	< 0,001
Male	8.436	68,4	8.033	68,0	403	76,8	1,52 (1,26;1,91)	
Age distribution (years)^b^								
10-17	450	3,7	444	3,8	6	1,2	1,00	< 0,001
18-39	5.389	44,2	5.281	45,2	108	20,8	1,51 (0,66;3,46)	0,327
40-59	4.081	33,5	3.893	33,4	188	36,2	3,57 (1,58;8,10)	0,002
60+	2.271	18,6	2.053	17,6	218	41,9	7,86 (3,47;17,80)	< 0,001
Race/skin color^b^								
White	2.553	21,2	2.442	21,1	111	21,9	1,00	< 0,001
Non-white	9.508	78,8	9.111	78,9	397	78,1	0,96 (0,77;1,19)	
Schooling^b^								
Complete or incomplete higher education	788	7,9	778	8,1	10	2,7	1,00	0,134
Complete elementary education and complete high school education	3.539	35,4	3.465	36,0	74	19,9	1,66 (0,86;3,23)	< 0,001
Incomplete elementary education and incomplete high school education	4.856	48,6	4.636	48,2	220	59,1	3,69 (1,95;6,99)	< 0,001
Illiterate	808	8,1	740	7,7	68	18,3	7,15 (3,65;13,99)	< 0,001
Street dweller^b^								
No	6.392	97,1	6.129	97,3	263	93,6	1,00	< 0,001
Yes	189	2,9	171	2,7	18	6,4	2,45 (1,49;4,49)	
Health professionals^b^								
No	6.467	98,1	6.185	98,1	282	99,6	1,00	< 0,001
Yes	124	1,9	123	1,9	1	0,4	0,18 (0,03;1,28)	
Immigrants^b^								
No	6.527	99,2	6.247	99,2	280	99,3	1,00	< 0,001
Yes	50	0,8	48	0,8	2	0,7	0,93 (0,23;3,84)	
Deprived of liberty^b^								
No	9.519	85,9	9.086	85,4	433	96,9	1,00	< 0,001
Yes	1.567	14,1	1.553	14,6	14	3,1	0,19 (0,11;0,323)	

a) RR: Relative Risk; 95%CI: 95% confidence interval; b) The difference in relation to the total number of cases (100%) corresponds to the number of cases that were unknown, left blank or filled in with data not corresponding to the tuberculosis Dictionary.

Regarding type of treatment, cases classified as retreatment showed greater association with the unfavorable outcome of death (RR: 1.34; 95%CI 1.04;1.72). Alcohol use also presented risk of death (RR: 1.73; 95%CI 1.39;2.17), as did tobacco use (RR: 1.54; 95%CI 1.18;2.01). Furthermore, diabetes also presented significant risk of death (RR: 1.58; 95%CI 1.17;2.15). Laboratory confirmation (sputum/other material smear microscopy, sputum/other material culture, rapid molecular test or histology with positive AFB) was shown to be a protective factor (RR: 0.59; 95%CI 0.49;0.70) (Table 2).

In the final model, the following remained independently associated with death due to tuberculosis: age group ≥ 60 years (RR: 7.70; 95%CI 1.91;31.04), illiteracy (RR: 4.50; 95%CI 1.60;12.66), incomplete elementary education and incomplete high school education (RR: 3.66; 95%CI 1.34;9.96), street dweller (RR: 2.41; 95%CI 1.34;4.35), male sex (RR: 1.48; 95%CI 1.04;2.09), alcohol use (RR: 1.45; 95%CI 1.04;2.02), tobacco use (RR: 1.32; 95% CI 0.98;1.77) and laboratory confirmation (RR: 0.68; 95%CI 0.52;0.93) (Table 3).

**Table 2:** Analysis of Relative Risks (RR) and respective Confidence Intervals (95%CI) relating to the clinical and behavioral characteristics of individuals with tuberculosis, by cases and deaths, Mato Grosso, Brazil, 2011-2020

Clinical and behavioral characteristics	Total n = 12.331		Tuberculosis cases					
			Non-deaths n = 11.806		Deaths n = 525		RR (95%CI)^a^	p-value
	N	%	N	%	N	%		
Treatment^b^								
Caso novo	10.725	88,4	10.289	88,5	436	85,2	1,00	< 0,001
Retratamento	1.413	11,6	1.337	11,5	76	14,8	1,34 (1,04;1,72)	
Observed treatment^b^								
Not observed	1.075	22,0	1.037	22,0	38	21,3	1,00	< 0,001
Observed	3.819	78,0	3.679	78,0	140	78,7	1,04 (0,72;1,50)	
Alcohol use^b^								
No	9.377	84,5	9.035	84,8	342	76,3	1,00	< 0,001
Yes	1.722	15,5	1.616	15,2	106	23,7	1,73 (1,39;2,17)	
Drug use^b^								
No	6.106	89,5	5.855	89,5	251	88,7	1,00	< 0,001
Yes	719	10,5	687	10,5	32	11,3	1,09 (0,75;1,58)	
Tobacco use^b^								
No	5.391	78,3	5.195	78,7	196	70,5	1,00	< 0,001
Yes	1.491	21,7	1.409	21,3	82	29,5	1,54 (1,18;2,01)	
Diabetes^b^								
No	10.153	92,6	9.755	92,8	398	89,0	1,00	0,003
Yes	807	7,4	758	7,2	49	11,0	1,58 (1,17;2,15)	
HIV^b^								
No	6.701	87,6	6.442	87,8	259	84,1	1,00	0,056
Yes	948	12,4	899	12,2	49	15,9	1,36 (0,99;1,85)	
Laboratory confirmation^b^								
No	5.807	47,1	5.494	46,5	313	59,6	1,00	< 0,001
Yes	6.524	52,9	6.312	53,5	212	40,4	0,59 (0,49;0,70)	

a) RR: Relative Risk; 95%CI: 95% confidence interval; b) The difference in relation to the total number of cases (100%) corresponds to the number of cases that were unknown, left blank or filled in with data not corresponding to the tuberculosis Dictionary.

**Table 3:** Analysis of crude and adjusted Relative Risks relating to the sociodemographic, socioeconomic, dwelling place, behavioral and clinical characteristics associated with tuberculosis deaths, state of Mato Grosso, Brazil, 2011-2020

	Total n = 12.331		Non-deaths n = 11.806		Deaths n = 525		Crude RR (95%CI)^a^	Adjusted RR (95%CI)^a^	p-value
	N	%	N	%	N	%			
Age group^b^									
10-17	450	3,7	444	3,8	6	1,2	1,00	1,00	
18-39	5.389	44,2	5.281	45,2	108	20,8	1,51 (0,66;3,46)	1,43 (0,35;5,87)	0,624
40-59	4.081	33,5	3.893	33,4	188	36,2	3,57 (1,58;8,10)	2,84 (0,70;11,54)	0,145
60+	2.271	18,6	2.053	17,6	218	41,9	7,86 (3,47;17,80)	7,70 (1,91;31,04)	0,004
Schooling^b^									
Higher education (complete or incomplete)	788	7,9	778	8,1	10	2,7	1,00	1,00	
Complete elementary education and complete high school education	3.539	35,4	3.465	36,0	74	19,9	1,66 (0,86;3,23)	2,72 (0,98;7,56)	0,054
Incomplete elementary education and incomplete high school education	4.856	48,6	4.636	48,2	220	59,1	3,69 (1,95;6,99)	3,66 (1,34;9,96)	0,011
Illiterate	808	8,1	740	7,7	68	18,3	7,15 (3,65;13,99)	4,50 (1,60;12,66)	0,054
Street dweller^b^									
No	6.392	97,1	6.129	97,3	263	93,6	1,00	1,00	0,003
Yes	189	2,9	171	2,7	18	6,4	2,45 (1,49;4,049)	2,41 (1,34;4,35)	
Alcohol use^b^									
No	9.377	84,5	9.035	84,8	342	76,3	1,00	1,00	0,027
Yes	1.722	15,5	1.616	15,2	106	23,7	1,73 (1,39;2,17)	1,45 (1,04;2,02)	
Laboratory confirmation^b^									
No	5.807	47,1	5.494	46,5	313	59,6	1,00	1,00	0,014
Yes	6.524	52,9	6.312	53,5	212	40,4	0,59 (0,49;0,70)	0,68 (0,52;0,93)	
Sex^b^									
Female	3.895	31,6	3.773	32,0	122	23,2	1,00	1,00	0,027
Male	8.436	68,4	8.033	68,0	403	76,8	1,52 (1,26;1,91)	1,48 (1,04;2,09)	
Tobacco use^b^									
No	5.391	78,3	5.195	78,7	196	70,5	1,00	1,00	0,259
Yes	1.491	21,7	1.409	21,3	82	29,5	1,54 (1,18;2,01)	1,32 (0,98;1,77)	

a) RR: Relative Risk; 95%CI: 95% confidence interval. b) A The difference in relation to the total number of cases (100%) corresponds to the number of cases that were unknown, left blank or filled in with data not corresponding to the tuberculosis Dictionary; Hosmer-Lemeshow test: p = 0.9619.

## DISCUSSION

In this study, being over 60 years old, having low levels of schooling, being a street dweller, alcohol use, being of the male sex and tobacco use were associated with death due to tuberculosis, while laboratory confirmation proved to be a protective factor in relation to the outcome. These results demonstrate that the risk factors associated with death due to tuberculosis corroborate the findings of other authors who assessed causes of death in people with the disease and identified factors most associated with death, as well as risk factors associated with treatment dropout in their respective study locations. ^(^ ^16^ ^)-(^ ^19^

Advanced age was a predisposing factor for death due to tuberculosis, with people over 60 years of age being more susceptible. On a national level, tuberculosis mortality mainly affects individuals over 45 years of age. In a study carried out in Rio de Janeiro, the authors analyzed the multiple causes of death in a cohort of patients reported as having tuberculosis between 2006 and 2008, whereby a higher frequency of deaths was found among the elderly. ^(^ ^16^ In addition to these, other studies have also found and reported association of older age groups with the outcome of death due to tuberculosis since the beginning of the 2000s in the states of São Paulo ^17^ and Rio de Janeiro, ^(^ ^18^ a fact that was also found more recently in indigenous populations in Peru, between 2015 and 2019. ^(^ ^19^

Population aging is a phenomenon that is occurring rapidly in Brazil and brings with it several aggravating factors in various aspects of a person’s life, such as physiological, biochemical and functional changes, which increase susceptibility to infections such as tuberculosis. ^(^ ^20^ Among the elderly, there is commonly a decline in cellular immune reactivity as well as the existence of multiple chronic diseases (multimorbidity), a reality that, in Brazil, is already present in a large part of the population over 50 years of age. ^(^ ^21^ Tuberculosis, in this case, can be more difficult to detect, as this population often presents other respiratory complications with similar symptoms. Furthermore, at this stage in life it is common for memory deficits and states of confusion to occur, these being factors that tend to cause treatment discontinuity and worsen cases, which end up leading to death. ^(^ ^22^

Still within the Brazilian context, there is considerable economic inequality. Although the country has considerable accumulated wealth, a large part of the population is subjected to a state of poverty. ^(^ ^23^ Income disparities and illiteracy rates are commonly associated with lower life expectancy in Brazil, and this scenario is no different in Mato Grosso state. Notwithstanding, the state has one of the highest GDP *per capita* in Brazil, coming in 11th place in 2019, with GDP per capita of R$ 21,435.36, HDI equal to 0.736 and Gini index equal to 0.461, a scenario that seems incompatible with outcomes associated with tuberculosis. ^(^ ^11^

Low levels of education, also identified by this study as a predisposing factor for death due to tuberculosis, reflect a combination of socioeconomic determinants that increases vulnerability to the disease and contributes to an increase in its incidence and unfavorable outcomes, as found by a study conducted in Rio de Janeiro from 2004 to 2006. ^(^ ^24^ There is another significant factor among these determinants: housing/dwelling conditions and sanitation. It is known that people living on the streets are more susceptible to tuberculosis precisely because of their precarious living conditions and limited access to health services. The disease even acts as a marker of social inequities in health. ^(^ ^25^

 This study also found that people of the male sex are more affected and are more associated with death due to tuberculosis in Mato Grosso, a fact that follows the pattern of the disease in terms of national and global figures. ^(^ ^1^ ^),(^ ^4^ According to the Ministry of Health’s trend study, out of the total number of new cases reported in Brazil between 2020 and 2022, 70% occurred in people of the male sex, these being those at greatest risk of becoming ill. ^(^ ^4^ Research conducted in Rondônia state, covering the period from 2010 to 2015, further reports that males tend not to seek treatment both due to the stigma it causes and the influence of work activities in their daily lives. ^(^ ^26^ Likewise, this population has the highest percentages of use of alcohol, tobacco and other drugs, which had already been identified in a study using national SINAN data, for the period 2007-2011, as factors related to treatment dropout and, consequently, also associated with death. ^(^ ^22^ ^),(^ ^27^

Almost half of the cases identified did not have laboratory confirmation. This may occur due to the natural condition of the disease itself, in relation to paucibacillary cases, in which sputum samples are negative, ^(^ ^28^ as well as being a reflection of health service organization, as shown in a study carried out in six municipalities in the Southeast, South and Northeast regions of Brazil between 2008 and 2009.^29^ On the other hand, the finding that people with laboratory confirmation of tuberculosis are at lower risk of death raises several interpretative possibilities. Individuals undergoing laboratory confirmation may have received earlier and more effective treatment, which would contribute to greater survival. The lack of specific studies on this association highlights the importance of future investigations to validate and better understand this phenomenon.

The results identified through the final Poisson regression model corroborate data from the literature and emphasize the importance of investing in policies capable of reducing inequalities and guaranteeing access and adequate assistance to people with tuberculosis. In this panorama, the WHO proposes the global End tuberculosis Strategy with the objective of eliminating the disease by the year 2035, addressing it as a multicausal phenomenon, prioritizing the most vulnerable populations and supporting their social protection and the communities where they live. ^(^ ^30^

The SINAN is the main instrument for collecting, analyzing and monitoring national data on tuberculosis, while the SIM was created to obtain mortality data on a regular and broad basis in Brazil. The use of these systems is extremely important, as they enable sociodemographic and epidemiological information to be obtained, supporting different levels of public service management in identifying risk factors and defining disease control priorities. Underreporting of cases and deaths on information systems hinders knowledge of the real epidemiological situation of tuberculosis, understanding of the dynamics of the disease in the territory, knowledge of socio-environmental risks, identification of the most vulnerable areas and planning of control measures. ^(^ ^12^

Among the limitations of this study is the impossibility of using the variable relating to observed treatment having been performed, or not, this being an important variable that can significantly influence the treatment outcome of patients with tuberculosis. Due to the large percentage of non-completion of this variable, we decided not to use it in the final model. Furthermore, another limitation to be mentioned is significant risk of death from tuberculosis among people living with HIV not being identified, which can be attributed to a series of factors. The database we used only included deaths for which tuberculosis was mentioned as the underlying cause. It is known that in cases of HIV co-infection, tuberculosis appears as the associated cause and HIV as the underlying cause. Another possible explanation would be the low rates of tuberculosis-HIV co-infection in the Midwest region, including the state of Mato Grosso. ^(^ ^4^

We therefore conclude that ages over 60 years, male sex, illiteracy, living on the streets, as well as alcohol and tobacco use, have a greater relationship with episodes of death due to tuberculosis. The results obtained through this study are relevant for the epidemiological characterization of death due to tuberculosis and for the identification of important social predictors, raising awareness among health services and guiding decision-making regarding actions relevant to controlling this disease, adapted to the reality of Mato Grosso. We suggest that a survival study be performed in order to understand the time factor, as well as a specific study on people with drug-resistant tuberculosis. In order to address the findings, health service managers and decision makers need to prioritize resources and formulate public policies targeting the social determinants identified.
