# Supplementary material for: Factors associated with deaths by tuberculosis in the state of Mato Grosso, 2011-2020: retrospective cohort study
Source: Epidemiol Serv Saude. 2024 Nov 11;33:e20231402. doi: 10.1590/S2237-96222024V33E20231402.EN (PMC11554292; doi:10.1590/S2237-96222024V33E20231402.EN)
Supplement: Translation

# Translation: Fatores associados aos óbitos por tuberculose no estado de Mato Grosso: estudo de coorte retrospectiva, 2011-2020

**DOI:** 10.1590/S2237-96222024V33E20231402.PT

**Authors:** Vanessa da Silva Lopes, Rodrigo de Macedo Couto, Alexandre Peron da Luz, Pãmela Rodrigues de Souza Silva, Jaqueline Costa Lima

**Affiliations:** 1 Universidade Federal de Mato Grosso, Faculdade de Enfermagem, Cuiabá, MT, Brasil; 2 Universidade Federal de São Paulo, Departamento de Medicina Preventiva, São Paulo, SP, Brasil; 3 Secretaria de Estado de Saúde, Cuiabá, MT, Brasil

**Keywords:** Tuberculose, Óbitos, Fatores de Risco, Estudo de Coorte Retrospectiva

## Abstract

**Objetivo:**

Investigar os fatores associados aos óbitos por tuberculose em Mato Grosso, no período de 2011 a 2020.

**Métodos:**

Coorte retrospectiva com dados obtidos pelo Sistema de Informação de Agravos de Notificação e pelo Sistema de Informação sobre Mortalidade. Os óbitos foram qualificados após relacionamento probabilístico e analisados por meio de regressão de Poisson.

**Resultados:**

Foram identificados 12.331 casos e 525 óbitos em 10 anos. Estiveram independentemente associados ao óbito: idade ≥ 60 anos (RR: 7,70; IC_95%_ 1,91;31,04), ensino fundamental e médio incompletos (RR: 3,66; IC95% 1,34;9,96), analfabetismo (RR: 4,50; IC_95%_ 1,60;12,66), população em situação de rua (RR: 2,41; IC_95%_ 1,34;4,35), uso de álcool (RR: 1,45; IC_95%_ 1,04;2,02), sexo masculino (RR: 1,48; IC_95%_ 1,04;2,09) e uso de tabaco (RR: 1,32; IC_95%_ 0,98;1,77). Confirmação laboratorial foi fator de proteção (RR: 0,68; IC_95%_ 0,52;0,93).

**Conclusão:**

Houve mais risco de morte em homens maiores de 60 anos, com baixa escolaridade, emsituação de vulnerabilidade e que usam álcool/tabaco.

## Contribuições do estudo

**Principais resultados:**

Foram identificados importantes preditores que elevam o risco de óbito por tuberculose: idade ≥ 60 anos, baixa escolaridade, população em situação de rua, uso de álcool, sexo masculino e uso de tabaco. A confirmação laboratorial foi fator de proteção.

**Implicações para os serviços:**

Sensibilização dos profissionais de saúde quanto aos fatores de risco associados aos óbitos por tuberculose no estado de Mato Grosso, especialmente quanto aos comportamentos de risco e à confirmação laboratorial da tuberculose.

**Perspectivas:**

Sugere-se estudo da sobrevida para compreender o fator tempo e estudo em pessoas com tuberculose drogarresistente. Gestores devem delinear políticas públicas voltadas aos determinantes observados.

## INTRODUÇÃO

A tuberculose é uma doença infecciosa e transmissível, cujo principal agente etiológico é o *Mycobacterium tuberculosis*, que acomete os pulmões e outros órgãos. Ela ocorre majoritariamente em países em desenvolvimento, mantendo íntima relação com populações em condições sociais, econômicas e culturais desfavorecidas, isto é, com a pobreza. ^(^ ^1^ ^)-(^ ^3^

A doença é considerada um problema de saúde mundial e, apesar de prevenível e curável, apresenta-se como uma das principais causas de óbito no mundo, ultrapassando até mesmo a síndrome da imunodeficiência adquirida (SIDA) e a malária. ^(^ ^1^ Somente no Brasil, que está entre os 30 países com maiores números de casos, em 2022, houve aproximadamente 78 mil notificações de casos novos e, em 2021, 5 mil mortes em sua decorrência.4 A redução desses números é particularmente a meta de interesse do Plano Nacional pelo Fim da Tuberculose como Problema de Saúde Pública, que estabelece uma redução de 90% na taxa de incidência e uma redução de 95% no número de mortes por tuberculose, ambas em comparação às taxas relatadas para 2015. ^(^ ^1^ ^),(^ ^4^ ^)^

O cenário descrito reflete a persistência da tuberculose como importante e desafiador problema no âmbito da saúde da população, e, considerando-se a produção social e multifatorial da doença, ^(^ ^5^ seus determinantes de mortalidade condicionam-se às diferentes características das dimensões demográfica, cultural e de desenvolvimento. Alguns estudos têm se preocupado em analisar e descrever esses fatores em vários estados e regiões do Brasil, permitindo comprovar sua variação em grau de relevância e ocorrência segundo as características do local de pesquisa. ^(^ ^6^ ^),(^ ^7^

Entretanto, a produção científica a respeito desse tema em Mato Grosso é incipiente e limitada, sendo mais comuns estudos ecológicos realizados em municípios prioritários específicos, como Rondonópolis e Barra do Garças, ou análises mais amplas do Centro-Oeste como um todo. ^(^ ^8^ ^)-(^ ^10^ Apesar de possuir um índice de desenvolvimento humano (IDH) considerado alto (0,736), ^(^ ^11^ o estado ainda possui alta endemicidade e mortalidade pela doença, tendo registrado em 2022 uma incidência de 31,8 casos por 100 mil habitantes e, em 2021, uma taxa de mortalidade de 2,0 óbitos por 100 mil habitantes. ^(^ ^4^ Esses números revelam um cenário que parece incongruente e que pode indicar a necessidade de se conhecerem os fatores determinantes específicos associados aos óbitos que atuam sobre essa população. 

A identificação desses fatores, proporcionada especialmente por essa investigação, é essencial para intensificar as medidas preventivas e a gestão dos serviços de saúde em todo o Brasil, uma vez que, com dados mais específicos, é possível promover o subsídio e o apoio às estratégias governamentais na elaboração de políticas públicas de saúde que sejam mais adaptadas às necessidades locais, a fim de atingir as metas do Plano Nacional pelo Fim da Tuberculose como Problema de Saúde Pública. 

Assim, este estudo teve como objetivo investigar os fatores associados aos óbitos por tuberculose em Mato Grosso, no período de 2011 a 2020.

## MÉTODOS

*Delineamento e local do estudo*

Estudo de coorte retrospectiva relacionado aos casos de tuberculose e à ocorrência do óbito pela doença na área territorial do estado de Mato Grosso, no período de 2011 a 2020, notificados no Sistema de Informação de Agravos de Notificação (Sinan) e no Sistema de Informação sobre Mortalidade (SIM). Os dados foram obtidos em fevereiro de 2023, por meio de dispositivo de armazenamento removível de dados, fornecidos pela Vigilância Epidemiológica da Secretaria Estadual de Saúde de Mato Grosso (SES-MT). Esses sistemas são de abrangência nacional e descentralizados para os municípios, que inserem os dados dos agravos destinados a compor os bancos estaduais e nacional do sistema. A notificação da tuberculose em Mato Grosso se tornou mais estruturada e eficiente a partir do final dos anos 1990 com a introdução do Sinan, melhorando significativamente a capacidade do estado de monitoramento e resposta. ^(^ ^12^

O estado de Mato Grosso, que possui 141 municípios, localiza-se na região Centro-Oeste do Brasil e ocupa uma área de 903.208,361 km². Sua população, segundo o último Censo de 2022, era de 3.658.813 habitantes. Sua densidade demográfica é de 4,05 hab./km^2^ e seu IDH, alto (0,736). ^(^ ^11^ Segundo o Cadastro Nacional de Estabelecimentos de Saúde (CNES), o estado conta com serviços distribuídos em todos os níveis de atenção, totalizando 9.103 estabelecimentos de saúde. Desses, 56% (n = 5.110) são unidades de serviços ambulatoriais; 12% (n = 1.113), unidades básicas de saúde (UBS) - com cobertura de 85% da população -; 8% (n = 745), unidades de serviço de apoio à diagnose e terapia, e cerca de 2% (n = 166) são hospitais. São oferecidos 9.341 leitos para internação hospitalar, sendo quase 73% (n = 6.778) deles ofertados pelo Sistema Único de Saúde. Dos leitos totais, 31% (n = 2.896) são clínicos (desses, 82% de clínica médica/geral); 28% (n = 2.609), cirúrgicos; 15% (n = 1.357), complementares, ou seja, de unidades de tratamento intensivo (UTIs) ou de cuidados intermediários; e 27%, (2.479) de outras especialidades. ^(^ ^13^

*Participantes*

A população de estudo foi composta de casos de tuberculose em pessoas de ambos os sexos com 10 anos ou mais. Foram excluídos aqueles assinalados com a categoria “mudança de diagnóstico”, presente na variável número 62 da ficha de acompanhamento de tuberculose, por não se tratar de casos da doença. Excluíram-se também aqueles com encerramento como “transferência” (refere-se ao paciente encaminhado para outro serviço para seguimento do tratamento) e que tiveram encerramentos por indicação de uso de esquemas de tratamento diferentes do esquema básico (conforme recomendação do Ministério da Saúde, esses casos devem ser encerrados no Sinan e notificados no Sistema de Informação de Tratamentos Especiais da Tuberculose - SITETB). O número de casos na área durante o período do estudo, submetidos a esses critérios, determinou o tamanho da amostra, cujo desfecho foram os óbitos e não óbitos para tuberculose.

*Variáveis, fonte de dados e mensuração*

A partir do Sinan, obtiveram-se informações: 

a) sociodemográficas: sexo (feminino e masculino); idade categorizada em faixas etárias (10 a 17 anos, 18 a 39 anos, 40 a 59 anos e 60 anos e mais); raça/cor da pele (branca e não branca, englobando pretos, pardos, indígenas e amarelos); escolaridade (ensino superior completo, ensino fundamental e médio completos, ensino fundamental e médio incompletos, analfabeto); pessoa em situação de rua (sim e não); profissional de saúde (sim e não); imigrante (sim e não); e privado de liberdade (sim e não). 

b) clínicas: tratamento (caso novo e retratamento); tratamento supervisionado (sim e não); diabetes (sim e não); HIV (sim e não); e confirmação laboratorial (sem confirmação e com confirmação). Esta última foi adicionada à análise após o agrupamento das variáveis referentes aos testes diagnósticos (baciloscopia de escarro, cultura e teste rápido molecular) em uma mesma variável, de forma que a realização de qualquer um dos três testes foi considerada suficiente para a confirmação laboratorial da tuberculose.

c) comportamentais: uso de álcool (sim e não); uso de drogas (sim e não); uso de tabaco (sim e não).

Os tipos de entrada, variável número 32 da ficha de notificação (“caso novo”, “não sabe” e “pós-óbito”), foram considerados casos novos, enquanto “recidiva” e “reingresso após abandono” foram recategorizados como retratamento. Foram considerados “óbitos por tuberculose” aqueles registrados como causa básica no SIM, e como “cura ou não óbito” aqueles correspondentes aos códigos de A15 a A19 da Classificação Internacional de Doenças (CID-10), no período de 2011 a 2020. 

Optou-se por não utilizar variáveis com percentual maior ou igual a 50% de valores ignorados ou em branco no modelo final. Na avaliação feita pelos autores, não foram identificadas variáveis como possíveis fontes de colinearidade.

*Análises estatísticas*

Os óbitos registrados no SIM foram utilizados para qualificação da informação registrada no Sinan, na variável “Situação de Encerramento”, campo 62 da ficha de acompanhamento. Utilizou-se, assim, o método de *linkage* probabilístico de registros, uma vez que as bases de dados utilizadas não continham variável identificadora única entre elas, por meio do *software* RecLink III (versão 3.1.6.3160). Esse método baseia-se na utilização conjunta de campos comuns de identificação presentes em ambos os bancos de dados, com o objetivo de identificar o quanto é provável que um par de registros corresponda ao mesmo indivíduo. ^(^ ^14^ No *software* Stata versão 16.1, inicialmente, foi realizada uma etapa de preparação das bases de dados por meio da padronização das variáveis de interesse (data de nascimento, nome do paciente, nome da mãe, sexo e município de residência). 

Posteriormente, utilizando-se o *software* RecLink III, foram criados blocos lógicos de registros em um processo composto por três etapas de blocagem. Algoritmos foram aplicados para realizar uma comparação aproximada de cadeias de caracteres, levando em consideração possíveis erros fonéticos e de digitação. Essa comparação foi realizada com base em informações, como nome, nome da mãe e data de nascimento. Foram calculados escores para resumir o grau geral de concordância entre registros pertencentes ao mesmo par. Na última etapa, ocorreu o pareamento (combinação) para estabelecer limiares que classificam os pares de registros como verdadeiros, não pares ou duvidosos. A revisão manual dos pares duvidosos foi conduzida por um único investigador, e, durante esse processo, a reclassificação desses pares em verdadeiros ou não verdadeiros seguiu critérios de desempate, como nome do paciente, data de nascimento, nome da mãe e município de residência. Os pares que permaneceram duvidosos após revisão manual foram classificados como não pertencentes ao mesmo indivíduo, adotando-se, portanto, uma abordagem mais conservadora.

Inicialmente, foi realizada uma análise descritiva com as distribuições das características da população de estudo, sendo os dados apresentados em tabelas com números absolutos e percentuais. Na sequência, foram estimados os riscos relativos (RRs) para estimar a magnitude da associação entre a variável de exposição e o óbito, indicando quantas vezes a ocorrência do óbito nos expostos é maior do que aquela entre os não expostos. Os RRs foram calculados pela razão entre a incidência do óbito em expostos e a incidência do óbito em não expostos. ^(^ ^15^ A significância estatística considerada foi de 5% e testada por meio do teste qui-quadrado, uma vez que as variáveis apresentavam distribuição categórica. 

Por fim, para análise dos fatores associados à ocorrência de óbito, utilizou-se modelo de regressão de Poisson, tomando-se como variável dependente óbito por tuberculose e como variáveis independentes, os potenciais fatores de risco. As estimativas dos RRs (bruta e ajustada) foram realizadas com os respectivos intervalos de confiança 95%. Para o modelo final de regressão de Poisson, foram selecionadas as variáveis com um valor de p < 0,20 na análise bruta. Sexo e faixa etária foram incluídos no modelo como variáveis de ajuste, baseadas na literatura sobre o tema. As variáveis preditoras foram adicionadas ao modelo uma por vez, começando com aquela que tinha o menor p-valor. Esse processo continuou até que todas as variáveis preditoras selecionadas tivessem sido testadas (método *forward*). A significância estatística (5%) das variáveis nos modelos foi avaliada pelo teste de Hosmer e Lemeshow. Para as análises estatísticas, utilizou-se o *software* Stata versão ^16^. 

*Aspectos éticos*

Este estudo foi aprovado em 18 de agosto de 2021 pelo Comitê de Ética em Pesquisa da Universidade Federal de Mato Grosso (Certificado de Apresentação de Apreciação Ética: 47657721.7.0000.8124) sob o parecer nº. 4.915.563.

## RESULTADOS

Do total inicial de 16.899 casos de tuberculose notificados no Sinan, referentes aos 10 anos do período analisado (2011 a 2020), foram excluídos: 86 registros duplicados (0,5%); 633 mudanças de diagnóstico (3,7%); 587 casos com idades até 9 anos (3,5%); 2 registros sem identificação de sexo (0,01%); 1.204 transferências (7,1%); 2.056 casos de drogarresistência, mudanças de esquema e falências (12,2%). Restaram, portanto, 12.331 (73%) notificações. Do banco do SIM, foram excluídos 8 óbitos com idades até 9 anos (1,1%), permanecendo 717 óbitos por tuberculose. 

Visando à qualificação dos óbitos, procedeu-se à técnica de *linkage* entre Sinan e SIM, na qual parearam-se 218 óbitos. Na sequência, foram somados 141 óbitos do Sinan não encontrados no SIM e 166 óbitos do SIM não encontrados no Sinan, totalizando 525 óbitos (Figura 1). Ao final da etapa de qualificação dos encerramentos, identificaram-se 12.331 casos de tuberculose, dos quais 525 (4,26%) evoluíram para óbito.

O maior risco relativo associado ao óbito por tuberculose esteve relacionado ao sexo masculino (RR: 1,52; IC_95%_ 1,26;1,91) (Tabela 1). Já no que se refere à faixa etária, obteve-se alto risco nas faixas etárias mais elevadas, apresentando efeito “dose-resposta”, ou seja, quanto maior a faixa etária, maior o risco de óbito (40 a 59 anos ‒ RR: 3,57; IC_95%_ 1,58;8,10; 60 anos ou mais ‒ RR: 7,86; IC_95%_ 3,47;17,80). Ainda, destacam-se os altos riscos relativos em indivíduos que tinham ensino fundamental e médio incompleto (RR: 3,69; IC_95%_ 1,95;6,99) ou eram analfabetos (RR: 7,15; IC_95%_ 3,65;13,99). No que concerne às populações especiais, pessoas em situação de rua apresentaram maior risco ao óbito (RR: 2,45; IC_95%_ 1,49;4,49) (Tabela 1).

**Figura 1:** Fluxograma do *linkage* entre os bancos de dados do Sistema de Informação de Agravos de Notificação (Sinan) e do Sistema de Informação sobre Mortalidade (SIM), Mato Grosso, 2011-2020

**Tabela 1:** Análise dos Riscos Relativos (RR) e respectivos Intervalos de Confiança (IC95%) das características demográficas, socioeconômicas e de moradia dos indivíduos e populações especiais com tuberculose segundo casos e óbitos, Mato Grosso, 2011-2020

Características demográficas, socioeconômicas e de moradia	Total n = 12.331		Casos de tuberculose					
			Não óbitos n = 11.806		Óbitos n = 525		RR (IC_95%_)^a^	p-valor
	N	%	N	%	N	%		
Sexo^b^								
Feminino	3.895	31,6	3.773	32,0	122	23,2	1,00	< 0,001
Masculino	8.436	68,4	8.033	68,0	403	76,8	1,52 (1,26;1,91)	
Distribuição etária (anos)^b^								
10 a 17 anos	450	3,7	444	3,8	6	1,2	1,00	< 0,001
18 a 39 anos	5.389	44,2	5.281	45,2	108	20,8	1,51 (0,66;3,46)	0,327
40 a 59 anos	4.081	33,5	3.893	33,4	188	36,2	3,57 (1,58;8,10)	0,002
60 anos e mais	2.271	18,6	2.053	17,6	218	41,9	7,86 (3,47;17,80)	< 0,001
Raça/cor da pele^b^								
Branca	2.553	21,2	2.442	21,1	111	21,9	1,00	< 0,001
Não branca	9.508	78,8	9.111	78,9	397	78,1	0,96 (0,77;1,19)	
Escolaridade^b^								
Ensino superior (completo ou incompleto)	788	7,9	778	8,1	10	2,7	1,00	0,134
Ensino fundamental completo e ensino médio completo	3.539	35,4	3.465	36,0	74	19,9	1,66 (0,86;3,23)	< 0,001
Ensino fundamental incompleto e ensino médio incompleto	4.856	48,6	4.636	48,2	220	59,1	3,69 (1,95;6,99)	< 0,001
Analfabeto	808	8,1	740	7,7	68	18,3	7,15 (3,65;13,99)	< 0,001
Em situação de rua^b^								
Não	6.392	97,1	6.129	97,3	263	93,6	1,00	< 0,001
Sim	189	2,9	171	2,7	18	6,4	2,45 (1,49;4,49)	
Profissionais da saúde^b^								
Não	6.467	98,1	6.185	98,1	282	99,6	1,00	< 0,001
Sim	124	1,9	123	1,9	1	0,4	0,18 (0,03;1,28)	
Imigrantes^b^								
Não	6.527	99,2	6.247	99,2	280	99,3	1,00	< 0,001
Sim	50	0,8	48	0,8	2	0,7	0,93 (0,23;3,84)	
Privados de liberdade^b^								
Não	9.519	85,9	9.086	85,4	433	96,9	1,00	< 0,001
Sim	1.567	14,1	1.553	14,6	14	3,1	0,19 (0,11;0,323)	

a) RR: Risco Relativo; IC95%: Intervalo de confiança de 95%; b) A diferença para o número total de casos (100%) corresponde ao número de casos ignorados, em branco ou com preenchimento sem correspondência no Dicionário de dados do agravo tuberculose.

Quanto ao tipo de tratamento, os casos classificados como retratamento apresentaram maior associação ao desfecho desfavorável de óbito (RR: 1,34; IC_95%_ 1,04;1,72). O uso de álcool também se apresentou como risco ao óbito (RR: 1,73; IC_95%_ 1,39;2,17), assim como o uso de tabaco (RR: 1,54; IC_95%_ 1,18;2,01). Ademais, o agravo diabetes também apresentou risco significativo de óbito (RR: 1,58; IC_95%_ 1,17;2,15). A confirmação laboratorial (baciloscopia de escarro/outro material, cultura de escarro/outro material, teste rápido molecular ou histologia com BAAR positivo) apresentou-se como fator de proteção (RR: 0,59; IC_95%_ 0,49;0,70) (Tabela 2).

No modelo final, permaneceram independentemente associados ao óbito por tuberculose: faixa etária ≥ 60 anos (RR: 7,70; IC_95%_ 1,91;31,04), analfabetismo (RR: 4,50; IC_95%_ 1,60;12,66), ensino fundamental incompleto e ensino médio incompleto (RR: 3,66; IC_95%_ 1,34;9,96), população em situação de rua (RR: 2,41; IC_95%_ 1,34;4,35), sexo masculino (RR: 1,48; IC_95%_ 1,04;2,09), uso de álcool (RR: 1,45; IC_95%_ 1,04;2,02), uso de tabaco (RR: 1,32; IC_95%_ 0,98;1,77) e confirmação laboratorial (RR: 0,68; IC_95%_ 0,52;0,93) (Tabela 3).

**Tabela 2:** Análise dos Riscos Relativos (RR) e respectivos Intervalos de Confiança (IC95%) das características clínicas e comportamentais dos indivíduos com tuberculose segundo casos e óbitos, Mato Grosso, 2011-2020

Características clínicas e comportamentais	Total n = 12.331		Casos de tuberculose					
			Não óbitos n = 11.806		Óbitos n = 525		RR (IC_95%_)^a^	p-valor
	N	%	N	%	N	%		
Tratamento^b^								
Caso novo	10.725	88,4	10.289	88,5	436	85,2	1,00	< 0,001
Retratamento	1.413	11,6	1.337	11,5	76	14,8	1,34 (1,04;1,72)	
Tratamento supervisionadoa^b^								
Sem supervisão	1.075	22,0	1.037	22,0	38	21,3	1,00	< 0,001
Com supervisão	3.819	78,0	3.679	78,0	140	78,7	1,04 (0,72;1,50)	
Álcool^b^								
Não	9.377	84,5	9.035	84,8	342	76,3	1,00	< 0,001
Sim	1.722	15,5	1.616	15,2	106	23,7	1,73 (1,39;2,17)
Drogas^b^								
Não	6.106	89,5	5.855	89,5	251	88,7	1,00	< 0,001
Sim	719	10,5	687	10,5	32	11,3	1,09 (0,75;1,58)
Tabaco^b^								
Não	5.391	78,3	5.195	78,7	196	70,5	1,00	< 0,001
Sim	1.491	21,7	1.409	21,3	82	29,5	1,54 (1,18;2,01)	
Diabetes^b^								
Não	10.153	92,6	9.755	92,8	398	89,0	1,00	0,003
Sim	807	7,4	758	7,2	49	11,0	1,58 (1,17;2,15)	
HIV^b^								
Não	6.701	87,6	6.442	87,8	259	84,1	1,00	0,056
Sim	948	12,4	899	12,2	49	15,9	1,36 (0,99;1,85)	
Confirmação laboratorial^b^								
Sem confirmação	5.807	47,1	5.494	46,5	313	59,6	1,00	< 0,001
Com confirmação	6.524	52,9	6.312	53,5	212	40,4	0,59 (0,49;0,70)	

a) RR: Risco Relativo; IC95%: Intervalo de confiança de 95%; b) A diferença para o número total de casos (100%) corresponde ao número de casos ignorados, em branco ou com preenchimento sem correspondência no Dicionário de dados do agravo tuberculose.

**Tabela 3:** Análise dos Riscos Relativos brutos e ajustados das características sociodemográficas, socioeconômicas, de moradia, comportamentais e clínicas associadas aos óbitos por tuberculose, estado de Mato Grosso, 2011-2020

	Total n = 12.331		Não óbitos n = 11.806		Óbitos n = 525		RR bruto (IC_95%_)^a^	RR ajustado (IC_95%_)^a^	p-valor
	N	%	N	%	N	%			
Faixa etária^b^									
10 a 17 anos	450	3,7	444	3,8	6	1,2	1,00	1,00	
18 a 39 anos	5.389	44,2	5.281	45,2	108	20,8	1,51 (0,66;3,46)	1,43 (0,35;5,87)	0,624
40 a 59 anos	4.081	33,5	3.893	33,4	188	36,2	3,57 (1,58;8,10)	2,84 (0,70;11,54)	0,145
60 anos e mais	2.271	18,6	2.053	17,6	218	41,9	7,86 (3,47;17,80)	7,70 (1,91;31,04)	0,004
Escolaridade^b^									
Ensino superior (completo ou incompleto)	788	7,9	778	8,1	10	2,7	1,00	1,00	
Ensino fundamental completo e ensino médio completo	3.539	35,4	3.465	36,0	74	19,9	1,66 (0,86;3,23)	2,72 (0,98;7,56)	0,054
Ensino fundamental incompleto e ensino médio incompleto	4.856	48,6	4.636	48,2	220	59,1	3,69 (1,95;6,99)	3,66 (1,34;9,96)	0,011
Analfabeto	808	8,1	740	7,7	68	18,3	7,15 (3,65;13,99)	4,50 (1,60;12,66)	0,054
População em situação de rua^b^				
Não	6.392	97,1	6.129	97,3	263	93,6	1,00	1,00	0,003
Sim	189	2,9	171	2,7	18	6,4	2,45 (1,49;4,049)	2,41 (1,34;4,35)	
Álcool^b^									
Não	9.377	84,5	9.035	84,8	342	76,3	1,00	1,00	0,027
Sim	1.722	15,5	1.616	15,2	106	23,7	1,73 (1,39;2,17)	1,45 (1,04;2,02)	
Confirmação laboratorial^b^									
Sem confirmação	5.807	47,1	5.494	46,5	313	59,6	1,00	1,00	0,014
Com confirmação	6.524	52,9	6.312	53,5	212	40,4	0,59 (0,49;0,70)	0,68 (0,52;0,93)	
Sexo^b^									
Feminino	3.895	31,6	3.773	32,0	122	23,2	1,00	1,00	0,027
Masculino	8.436	68,4	8.033	68,0	403	76,8	1,52 (1,26;1,91)	1,48 (1,04;2,09)	
Tabaco^b^									
Não	5.391	78,3	5.195	78,7	196	70,5	1,00	1,00	0,259
Sim	1.491	21,7	1.409	21,3	82	29,5	1,54 (1,18;2,01)	1,32 (0,98;1,77)	

a) RR: Risco Relativo; IC95%: Intervalo de confiança de 95%. b) A diferença para o número total de casos (100%) corresponde ao número de casos ignorados, em branco ou com preenchimento sem correspondência no Dicionário de dados do agravo tuberculose; Teste de Hosmer e Lemeshow: p = 0,9619.

## DISCUSSÃO

Neste estudo, idade acima de 60 anos, baixa escolaridade, população em situação de rua, uso de álcool, sexo e uso de tabaco foram associados ao óbito por tuberculose e a confirmação laboratorial se revelou como fator protetor em relação ao desfecho. Tais resultados demonstram que os fatores de risco associados ao óbito por tuberculose corroboram os achados de outros autores que avaliaram causas de óbitos em pessoas com tuberculose e identificaram fatores mais associados a essa evolução, bem como fatores de risco associados ao abandono do tratamento em seus respectivos locais de estudo. ^(^ ^16^ ^)-(^ ^19^

A idade avançada foi um fator predisponente de óbito por tuberculose, sendo as pessoas acima de 60 anos mais suscetíveis. No cenário nacional, a mortalidade por tuberculose atinge principalmente os indivíduos acima de 45 anos de idade. Em um estudo realizado no Rio de Janeiro, os autores analisaram as causas múltiplas de morte de uma coorte de pacientes notificados com tuberculose entre 2006 e 2008, sendo observada maior frequência de óbitos nos idosos. ^(^ ^16^ Além desses, outros estudos também têm encontrado e relatado associação de faixas etárias mais elevadas com o desfecho óbito por tuberculose, desde o início dos anos 2000, nos estados de São Paulo ^17^ e do Rio de Janeiro, ^(^ ^18^ fato que também foi constatado mais recentemente em populações indígenas do Peru, entre 2015 e 2019. ^(^ ^19^

O envelhecimento populacional é um fenômeno que ocorre de forma acelerada no país e traz consigo diversos agravantes em variados aspectos do indivíduo, como alterações fisiológicas, bioquímicas e funcionais, que acentuam a suscetibilidade a infecções como a tuberculose. ^(^ ^20^ Em idosos, é corrente o declínio da reatividade imune celular e a existência de múltiplas doenças crônicas (multimorbidade), realidade que, no Brasil, já está presente em grande parte da população acima de 50 anos. ^(^ ^21^ A tuberculose, nesse caso, pode ser mais difícil de ser detectada, pois essa população frequentemente apresenta outras complicações respiratórias com sintomas semelhantes. Além disso, nessa fase é comum a ocorrência de déficit de memória e estados de confusão, fatores que tendem a gerar descontinuidade no tratamento e a agravar os casos, que acabam evoluindo para óbito. ^(^ ^22^

Ainda no contexto brasileiro, nota-se uma considerável desigualdade econômica. Embora o país tenha grande riqueza acumulada, grande parte da população está submetida a um estado de pobreza. ^(^ ^23^ Disparidades de renda e taxas de analfabetismo estão comumente associadas a uma menor expectativa de vida no Brasil, e esse cenário não é diferente em Mato Grosso. No entanto, o estado apresenta um dos maiores Produtos Internos Brutos per capita do país, ocupando, em 2019, a 11ª posição, com um Produto Interno Bruto *per capita* de R$ 21.435,36, IDH de 0,736 e índice de Gini de 0,461, cenário que parece incompatível com os desfechos associados à tuberculose. ^(^ ^11^

O baixo nível de escolaridade, também apontado por este estudo como fator predisponente de óbito por tuberculose, reverbera um combinado de determinantes socioeconômicos que aumenta a vulnerabilidade à doença e contribui com o aumento da sua incidência e de seus desfechos desfavoráveis, como constatado por um estudo realizado no Rio de Janeiro de 2004 a 2006. ^(^ ^24^ Dentro desses determinantes está outro significativo fator: as condições de habitação/moradia e saneamento. Sabe-se que pessoas que vivem em situação de rua estão mais susceptíveis à tuberculose, justamente pelas condições precárias de vida e pelo acesso limitado aos serviços de saúde. A doença, inclusive, atua como marcador de iniquidades sociais em saúde. ^(^ ^25^

 Com este estudo, observou-se ainda que pessoas do sexo masculino são mais acometidas e estão mais associadas ao óbito por tuberculose em Mato Grosso, fato que segue o padrão da doença em números nacionais e globais. ^(^ ^1^ ^),(^ ^4^ De acordo com estudo de tendência do Ministério da Saúde, do total de casos novos notificados no Brasil entre 2020 e 2022, 70% ocorreram em pessoas do sexo masculino, sendo elas as que apresentam maior risco de adoecimento. ^(^ ^4^ Pesquisa realizada em Rondônia, no período de 2010 a 2015, ainda descreve que a população masculina tende a se distanciar do tratamento tanto pelo estigma gerado quanto pela influência das atividades laborais em seu cotidiano. ^(^ ^26^ Do mesmo modo, essa é a população com maiores percentuais de uso de álcool, tabaco e outras drogas, o que já havia sido apontado em estudo com dados nacionais do Sinan, de 2007 a 2011, como fatores relacionados ao abandono do tratamento e, consequentemente, associados também ao óbito. ^(^ ^22^ ^),(^ ^27^

Quase metade dos casos identificados não apresentaram confirmação laboratorial. Isso pode ocorrer pela própria condição natural da doença, com os casos paucibacilares, em que as amostras de escarro se encontram negativas, ^(^ ^28^ assim como pode ser reflexo da organização dos serviços de saúde, como apontou estudo realizado em seis municípios das regiões Sudeste, Sul e Nordeste do Brasil entre 2008 e 2009. ^(^ ^29^ Por outro lado, a observação de que pessoas com confirmação laboratorial de tuberculose apresentam menor risco de óbito suscita diversas possibilidades interpretativas. Indivíduos submetidos à confirmação laboratorial podem ter recebido tratamento mais precoce e eficaz, o que contribuiria para uma maior sobrevida. A falta de estudos específicos sobre essa associação destaca a importância de futuras investigações para validar e compreender melhor esse fenômeno.

Os resultados identificados por meio do modelo final de regressão de Poisson corroboram dados da literatura e enfatizam a importância de se investir em políticas capazes de reduzir as desigualdades e garantir o acesso e a adequada assistência a pessoas com tuberculose. Nesse panorama, a OMS propõe a estratégia global End tuberculose Strategy, com o objetivo de eliminar a doença até o ano de 2035, de modo a enfrentá-la como fenômeno multicausal, priorizando populações mais vulneráveis e apoiando a proteção social delas e das comunidades onde estão inseridas. ^(^ ^30^

O Sinan é o principal instrumento para coleta, análise e monitoramento dos dados nacionais acerca da tuberculose, e o SIM foi criado para a obtenção de dados de mortalidade de forma regular e ampla no Brasil. A utilização desses sistemas é de extrema relevância, pois eles permitem a obtenção de informações sociodemográficas e epidemiológicas, subsidiando diferentes esferas da gestão pública na identificação de fatores de risco e na definição de prioridades no controle da doença. A subnotificação de casos e óbitos, nos sistemas de informações, prejudica o conhecimento da real situação epidemiológica da tuberculose, o entendimento da dinâmica do agravo no território, o conhecimento de riscos socioambientais, a identificação de áreas mais vulneráveis e o planejamento de medidas de controle. ^(^ ^12^

Entre as limitações deste estudo, cita-se a impossibilidade de uso da variável “tratamento supervisionado efetivado”, uma importante variável que pode influenciar significativamente no desfecho de tratamento dos pacientes com tuberculose. Devido ao grande percentual de não preenchimento, optou-se por não a utilizar no modelo final. Além disso, outra limitação a ser mencionada é a não identificação de um risco significativo de óbito por tuberculose em indivíduos vivendo com HIV, o que pode ser atribuído a uma série de fatores. A base de dados utilizada contemplava somente óbitos com menção de tuberculose como causa básica. Sabe-se que, em casos de coinfecção com HIV, a tuberculose aparece como causa associada e o HIV como a causa básica. Outra possível explicação seriam as baixas taxas de coinfecção tuberculose-HIV na região Centro-Oeste, incluindo o estado de Mato Grosso. ^(^ ^4^

Conclui-se, portanto, que idades acima de 60 anos, sexo masculino, analfabetismo, população em situação de rua, bem como uso de álcool e tabaco possuem maior relação com episódios de óbito por tuberculose. Os resultados obtidos por meio deste estudo são relevantes para a caracterização epidemiológica do óbito por tuberculose e para a identificação de importantes preditores sociais, sensibilizando os serviços e norteando a tomada de decisão quanto às ações pertinentes ao controle desse agravo, adaptadas à realidade de Mato Grosso. Sugere-se estudo da sobrevida para a compreensão do fator tempo, assim como estudo específico em pessoas com TB drogarresistente. Para a implementação dos achados, gestores e tomadores de decisão devem priorizar recursos e delinear políticas públicas voltadas para os determinantes sociais identificados.
